# Underreporting of Dengue-4 in Brazil Due to Low Sensitivity of the NS1 Ag Test in Routine Control Programs

**DOI:** 10.1371/journal.pone.0064056

**Published:** 2013-05-23

**Authors:** Vanessa Ramos Faria Sea, Ana Cecília Ribeiro Cruz, Ricardo Q. Gurgel, Bruno Tardelli Diniz Nunes, Eliana Vieira Pinto Silva, Silvio S. Dolabella, Roseli La Corte dos Santos

**Affiliations:** 1 Universidade Federal de Sergipe, Aracaju, Sergipe, Brazil; 2 Instituto Evandro Chagas, Belém, Brazil; 3 Universidade do Estado do Pará, Belém, Para, Brazil; Northeast Agricultural University, China

## Abstract

We have identified fifty-eight samples that were positive for Dengue-4 among 119 samples with negative diagnoses for dengue via the Platelia™ dengue NS1 Ag in Aracaju, State of Sergipe, Brazil. We determined that the low sensitivity of the NS1 Ag test could be related to secondary dengue infections in the studied population. Therefore, we concluded that the sensitivity and specificity of the Platelia™ dengue NS1 Ag test as a screening method for monitoring circulating dengue serotypes must be reevaluated. In addition, regional endo-epidemic profiles should also be considered due to the prevalence of secondary responses.

## Introduction

Dengue is considered the most important of the arboviruses affecting humans. It is transmitted mainly by the mosquito *Aedes aegypti* and has four known serotypes: DENV-1, DENV-2, DENV-3 and DENV-4. Approximately 50 million people in over 100 nations on all continents (except Europe) are infected annually [1].

The first dengue epidemic that was documented in both the clinic and laboratory in Brazil occurred in 1981 in Boa Vista, State of Roraima, and DENV-1 and DENV-4 were identified [2]. In 1990, DENV-2 was also detected in Rio de Janeiro and was responsible for an extensive outbreak in the country and for the emergence of severe cases [3], [4]. However, the greatest epidemic of dengue in Brazil occurred in 2002, when almost 800,000 cases of the disease were reported, most of them DENV-3 [5]. In 2010, DENV-4 was detected in Boa Vista [6].

Since 2009, the NS1 antigen-capture ELISA test has been implemented by the Health Ministry in the Public Health laboratories network. Patients arriving for care within five days of the onset of disease symptoms are screened for viral isolation in health care sentinel units and, thus, provide early detection of circulating serotypes in a given area [7].

**Figure 1:**
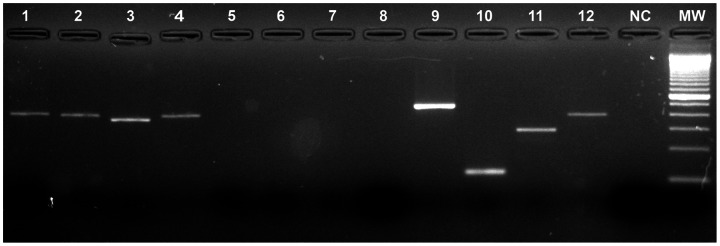
Reverse transcriptase-semi-nested-polymerase chain reaction (RT-PCR) product in 2% agarose gel stained with syber safe. Lines 1–4: positive samples; 5–8: negative samples; 9–12: positive control for DENV-1, DENV-2, DENV-3 and DENV-4; NC: negative control; MW: molecular weight marker.

In Sergipe state, routine NS1 Ag test detection protocol is applied to patients in the sentinel units according to Health Ministry determinations. Blood is collected and send to the Central Laboratory (Laboratório Central - LACEN/SE) for the Platelia™ test. Positive biological samples are then forwarded to the reference laboratory in Rio de Janeiro for viral isolation. Negative samples are stored in a −80°C and for this study were provided to our group in order to evaluate the presence of other arbovirus.

## Methods

### Ethics Statement

This study followed the ethical principles according to the Helsinki Declaration and was approved by the Research Ethics Committee of the Federal University of Sergipe (02872312.7.0000.0058). The samples analyzed were derived from routine diagnosis for dengue fever conducted by the Central Laboratory of Public Health (LACEN). Patients were asked and provided their oral consent to enter in the Ministry of Health protocol of diagnosis and isolation of serotype dengue virus. They were all informed that the serum was kept for further investigations of the circulation of other arboviruses and they were asked to return after 14 days for serological diagnosis. As that step was part of the routine of the dengue control program, there was not written term of consent available. Ethics committee dispensed patient’s written informed consent after we presented signed agreement by the authority responsible for the custody of the samples. Confidentiality of the identity and results was maintained.

### Study

To investigate the co-circulation of arboviruses in the State of Sergipe, blood samples negative for the NS1 antigen test from suspected dengue patients were requested from the Central Laboratory of Public Health. Platelia Dengue NS1 Ag test is a one-step sandwich-format microplate enzyme immunoassay for detecting dengue virus NS1 antigen. Tests were carried out on samples of serum or blood from patients and controls according to the manufacturer’s recommendations. Before being included in the protocol for the detection of other arboviruses these negative blood samples were initially evaluated by semi-nested RT-PCR for dengue [8] at the Evandro Chagas Institute (Instituto Evandro Chagas – IEC) to exclude possible dengue cases that might not have been detected by NS1 antigen-capture ELISA.

In total, 119 samples of serum or blood from symptomatic patients collected between September 2011 and February 2012 were obtained, and sample RNA was extracted using TRIzol® Plus reagent (Invitrogen™, California, US) according to the manufacturers’ instructions. Target viral RNA was converted to a DNA copy (cDNA) prior to enzymatic DNA amplification by use of reverse transcriptase (RT - MMLV/Invitrogen™) and the dengue virus downstream consensus primer (D2), homologous to the genomic RNA of the four serotypes. Subsequent *Taq* polymerase recombinant (Invitrogen™^,^ California, US) amplification was performed on the resulting cDNA with the upstream dengue virus consensus primer (D1). All relevant aspects of the RT-PCR and semi-nested-PCR (MgCl_2_, specific primers, RT, Taq polymerase, number of cycles, and annealing temperature) were as described by Lanciotti *et al.* (1992) [8]. A genomic fragment of 511 base pairs (bp) that corresponds to all DENV was obtained, followed by a semi nested PCR using specific primers for each of the four DENV serotypes. Samples were considered positive when DNA fragments measuring 482 bp (DENV-1), 392 bp (DENV-4), 290 bp (DENV-3), or 119 bp (DENV-2) were obtained. The semi-nested RT-PCR products were analyzed on a 3% agarose gel stained with SYBR Safe DNA gel stain (Invitrogen, USA). Suspensions of 1∶10 prepared in Leibowitz medium with 2% fetal bovine serum from positives samples were inoculated on C6/36 cells culture grown in a Leibowitz medium (L-15, Invitrogen, California, US). After 10 days each sample was submitted to indirect immunofluorescence assays (IFA) using monoclonal antibodies against the four dengue virus serotypes as previously described by Gubler *et al.* (1984) [9]. Cultures of uninfected cells were used as negative controls.

## Results and Discussion

When processing the samples, 58 (48.7%) samples were unexpectedly found to be positive for DENV-4 (Fig). No other serotype was detected. Of the 58 positive samples, 33 (56.9%) were from the capital city, Aracaju, while two (3.4%) were from the municipality of Itabaiana, and 23(39.7%) samples were from the municipality of Simão Dias; these two municipalities are 51 and 101 km from the capital, respectively. Positive samples were subjected to viral isolation in *Aedes albopictus* cells, clone C6/36s, to confirm the diagnosis, and 31 (53.5%) were positive for DENV-4.

The DENV-4 serotype was detected for the first time in Brazil in 1982 during a dengue outbreak in the north of the country, and since then, no new cases of DENV-4 were detected until 2010 [6]. In Sergipe, DENV-4 was not reported before March 2012, but, when we analyzed blood samples that were negative for dengue by the Platelia™ NS1 Ag test collected between September 2011 and February 2012, we found that 48.7% were positive for DENV-4 by semi-nested RT-PCR. These results suggest that the Platelia™ NS1 Ag test has a low sensitivity for the DENV-4 serotype. These findings corroborate those of Hang et al. (2009) [10], who demonstrated that NS1 Ag had variable sensibility to the different Dengue serotypes, although not evaluating Dengue 4 cases. The simultaneous presence of IgG, which suggests a secondary response and low viremia in the analyzed samples, should be considered as the factor that contributes to the low sensitivity of the NS1 Ag test for the examined samples [10]. The Platelia™ NS1 Ag test was also assessed in a multicenter study and showed overall sensitivities ranging from 36% (Central America) to 88% (Asian countries) depending on the serotype and the number of days since the onset of symptoms. In a Cuban study, when evaluations were conducted based on serotype, DENV-4 showed a sensitivity of 79% (95%CI = 67–91); however, few samples were analyzed [11]. In Brazil, the sensitivity was 83.6% for confirmed positive samples for DENV-1, DENV-2, and DENV-3, and better results were obtained using samples obtained up to the fifth day in the viremic phase [12]. However, in the above mentioned study, no correlation between sensitivity and specificity could be determined for DENV-4, as this serotype was not yet circulating in the country.

The low sensitivity of the NS1 Ag test should be considered in relation to secondary dengue infections in the studied population [12], [13], as the state of Sergipe can be considered endemic for dengue. In Sergipe, the median incidence has been 195 cases per 100,000 inhabitants (22.9–1620.9) over the past 16 years, and successive outbreaks involving the circulation of the other three serotypes have occurred since 1996 [14].

Although the implementation of the NS1 Ag test for viremic sample screening has efficiently increased the viral isolation percentage and permitted the identification of the circulating serotypes in Brazil [7], the findings of the present study strongly suggest that DENV-4 was previously circulating with a lower prevalence and was not being detected by the NS1 Ag test. This hypothesis may explain the speed with which DENV-4 has spread across the country since its detection in northern Brazil in 2010, in the south and southeast in early 2011, and even in small municipalities of the northeastern region in mid-2011, as observed in the present study.

Most likely due to the low sensitivity of the Platelia™ NS1 Ag test for DENV-4, there was a delay in detection and an underreporting of DENV-4 dengue cases in Brazil. As this serotype has been increasingly detected in many regions [7], the low sensitivity may have resulted in the absence of detected DENV-4 dengue cases and, obviously, in underreporting. Therefore, we conclude that the sensitivity, specificity and accuracy of the Platelia™ NS1 Ag test must be reevaluated as a screening method for monitoring circulating dengue serotypes and that regional endo-epidemic profiles and the high incidence of secondary responses should be taken into account.
